# Predicting intensive care need for COVID-19 patients using deep learning on chest radiography

**DOI:** 10.1117/1.JMI.10.4.044504

**Published:** 2023-08-21

**Authors:** Hui Li, Karen Drukker, Qiyuan Hu, Heather M. Whitney, Jordan D. Fuhrman, Maryellen L. Giger

**Affiliations:** The University of Chicago, Department of Radiology, Chicago, Illinois, United States

**Keywords:** COVID-19, artificial intelligence, machine learning, chest X-ray radiography

## Abstract

**Purpose:**

Image-based prediction of coronavirus disease 2019 (COVID-19) severity and resource needs can be an important means to address the COVID-19 pandemic. In this study, we propose an artificial intelligence/machine learning (AI/ML) COVID-19 prognosis method to predict patients’ needs for intensive care by analyzing chest X-ray radiography (CXR) images using deep learning.

**Approach:**

The dataset consisted of 8357 CXR exams from 5046 COVID-19–positive patients as confirmed by reverse transcription polymerase chain reaction (RT-PCR) tests for the SARS-CoV-2 virus with a training/validation/test split of 64%/16%/20% on a by patient level. Our model involved a DenseNet121 network with a sequential transfer learning technique employed to train on a sequence of gradually more specific and complex tasks: (1) fine-tuning a model pretrained on ImageNet using a previously established CXR dataset with a broad spectrum of pathologies; (2) refining on another established dataset to detect pneumonia; and (3) fine-tuning using our in-house training/validation datasets to predict patients’ needs for intensive care within 24, 48, 72, and 96 h following the CXR exams. The classification performances were evaluated on our independent test set (CXR exams of 1048 patients) using the area under the receiver operating characteristic curve (AUC) as the figure of merit in the task of distinguishing between those COVID-19–positive patients who required intensive care following the imaging exam and those who did not.

**Results:**

Our proposed AI/ML model achieved an AUC (95% confidence interval) of 0.78 (0.74, 0.81) when predicting the need for intensive care 24 h in advance, and at least 0.76 (0.73, 0.80) for 48 h or more in advance using predictions based on the AI prognostic marker derived from CXR images.

**Conclusions:**

This AI/ML prediction model for patients’ needs for intensive care has the potential to support both clinical decision-making and resource management.

## 1 Introduction

The coronavirus disease 2019 (COVID-19) is an ongoing pandemic caused by severe acute respiratory syndrome coronavirus 2, which was first reported in late 2019. As of June 28, 2023, there have been 767,518,723 confirmed cases of COVID-19, including 6,947,192 deaths.^1^ The reverse transcription polymerase chain reaction (RT-PCR) is the reference standard currently used for COVID-19 disease diagnosis. In addition, clinical assessment^2^ and multimodality medical imaging^3^ are also used in disease diagnosis and patient management.

Artificial intelligence/machine learning (AI/ML), including deep learning, has been applied in medical imaging and radiation therapy for several decades.^4^^–^^8^ Accordingly, various studies have been reported using AI/ML on medical imaging for COVID-19 disease. AI/ML algorithms have been developed to differentiate COVID-19 pneumonia from non-COVID-19 pneumonia when RT-PCR is not readily available.^9^^–^^12^ Various AI/ML methods have been developed to assess the severity/extent of disease^13^^–^^16^ and predict the prognosis of the disease,^17^ as well as for patient management in therapeutic treatment planning and monitoring patients’ response.^13^^,^^18^ Image-based studies of long-term COVID-19 effects on other organs, including the heart and brain, are also underway.^19^

Accurate prognosis prediction for COVID-19 patients is crucial not only for implementing appropriate treatment for individual patients, but also for optimizing medical resource allocation during the pandemic. Chest X-ray radiography (CXR) is recommended for triaging at patient presentation and disease monitoring due to its ease of use, relatively low cost, wide availability, and portability.^3^^,^^20^^,^^21^ Characteristics such as bilateral lower lobe consolidations, ground glass opacities, peripheral air space opacities, and diffuse air space disease on CXR have been related to COVID-19.^22^^,^^23^ However, the non-specificity of these features to COVID-19 and the shortage of radiological expertise in some resource-strained healthcare systems during a pandemic make precise image assessments challenging.

There are various studies in intensive care unit (ICU) requirement prediction for COVID-19 patients using AI/ML.^24^^–^^32^ Those predicting models are based on clinical data, laboratory test results, comorbidity data, genetic data, and imaging data. Heo et al.^24^ performed the logistic regression analysis to predict ICU admission status using clinical, radiological, and laboratory variables. An area under the curve (AUC) value of 0.880 was obtained from an integer-based scoring system using seven selected features. Asteris et al.^26^ developed an artificial neural network (ANN) model based on complement-related genetic variants, age, and gender to predict ICU admission. They reported an accuracy of 89.47% in predicting COVID-19 severity using a sample of 133 patients with the developed ANN model. Chieregato et al.^27^ built a hybrid ML/deep learning model for ICU prediction using CT images and clinical data from 558 patients with high sensitivity and specificity as well as SHapley Additive exPlanations (SHAP) values for each individual feature corresponding to the importance of each feature in the prediction model to increase the interpretability of the model.

Training a deep learning model from scratch in the medical imaging field is a challenging task since it requires large well-curated medical imaging datasets with annotations provided by medical professionals. Due to the nature of medical imaging datasets, most with necessary human-delineated annotations are small in size. Therefore, a technique called “transfer learning” has emerged to bridge this gap and has been applied in medical imaging analysis.^33^ In these situations, deep learning models pretrained on nonmedical image datasets or medical image datasets from either a different imaging modality or same imaging modality but for different clinical tasks are fine-tuned with a relatively small medical imaging dataset for clinical decision-making tasks.^33^^–^^39^ For example, Antropova et al.^34^ applied transfer learning on three different imaging modalities to extract deep features and fused them with human engineered radiomic features for the diagnostic classification of breast tumors, with results demonstrating statistically significant improved classification performance as compared to previous developed computer-aided diagnosis methods. Huang et al.^35^ applied deep transfer learning to identify possible disease on CXR images for multilabel classification task with improved prediction capacities. Samala et al.^36^ performed a multi-stage transfer learning for the classification of malignant and benign masses in digital breast tomosynthesis images and reported improved classification performance.

The purpose of our study was to develop an AI/ML COVID-19 prognosis method to predict patients’ need for intensive care by analyzing CXR images of COVID-19–positive patients using deep learning with a sequential transfer learning strategy.

## 2 Materials and Methods

### 2.1 Dataset

A limited deidentified dataset was retrospectively collected from our institution under a Health Insurance Portability and Accountability Act (HIPAA)-compliant, Institutional Review Board-approved protocol during the COVID-19 outbreak, consisting of CXR exams acquired between Feb 27, 2020 and January 21, 2022. From patients who underwent the RT-PCR test for the SARS-CoV-2 virus, CXR exams and clinical data were collected after the initial RT-PCR tests. The clinical data used in this study were last updated on March 13, 2022. In this study, intensive care is defined as intubation (invasive mechanical ventilation) and/or ICU admission. We assumed that all patients who needed intensive care were admitted without delay during this study period. Chest radiographs of two groups of COVID-19–positive patients were included in this study. One group consisted of COVID-19–positive patients who needed intubation or ICU support. The other group consisted of COVID-19–positive patients who were not admitted to ICU and did not need intubation following their COVID-19 diagnosis. The intubation or ICU admission information was extracted from patients’ clinical information and radiology reports. The ICU admission or intubation time was compared with the imaging exam time to determine the time elapsed between imaging and any potential subsequent intubation or ICU admission event. For example, if the CXR exam was obtained within the 24 h prior to ICU admission or intubation, then the ICU admission status for 24, 48, 72, and 96 h would all be true; if the CXR exam was obtained less than 48 h but more than 24 h prior to the intubation/ICU admission event, then the 24-h status would be false, while the 48, 72, and 96 statuses would be true. For a patient without an intubation or ICU admission event, all statuses would be false. Only images acquired after a positive RT-PCR were included, and images obtained after ICU admission or intubation were excluded. Ultimately, the dataset for this study consisted of 8357 CXR images from 5046 COVID-19–positive patients. Patient demographics are summarized in Table 1. Patients were largely unvaccinated, with only 16% having received one or more vaccinations against COVID-19 at the time of imaging.

**Table 1:** Patient demographics of the COVID-19 dataset. Age is reported in years as mean ± standard deviation.

Dataset	Entire dataset		Training set		Validation set		Test set	
By patients	Number of patients	Age (years)	Number of patients	Age (years)	Number of patients	Age (years)	Number of patients	Age (years)
Number of patients	5046	54.5±19.1	3181 (63.0%)	54.3±19.0	817 (16.2%)	55.7±19.2	1048 (20.8%)	54.2±19.3
**Sex**								
Female	2833 (56.1%)	54.7±19.7	1780 (56.0%)	54.6±19.6	453 (55.4%)	55.1±19.7	600 (57.3%)	54.4±19.9
Male	2213 (43.9%)	54.4±18.3	1401 (44.0%)	54.0±18.2	364 (44.6%)	56.5±18.5	448 (42.7%)	54.0±18.5
**Race**								
American Indian or Alaska Native	9 (0.2%)		4 (0.1%)		0 (0.0%)		5 (0.5%)	
Asian/Mideast Indian	44 (0.9%)		28 (0.9%)		10 (1.2%)		6 (0.6%)	
Black/African-American	4241 (84.0%)		2687 (84.5%)		666 (81.5%)		888 (84.7%)	
More than one race	198 (3.9%)		120 (3.8%)		37 (4.5%)		41 (3.9%)	
Native Hawaiian/other Pacific Islander	4 (0.1%)		2 (0.1%)		0 (0.0%)		2 (0.2%)	
White	464 (9.2%)		278 (8.7%)		92 (11.3%)		94 (9.0%)	
Unknown/patient declined	86 (1.7%)		62 (1.9%)		12 (1.5%)		12 (1.1%)	
**Ethnicity**								
Hispanic or Latino	271 (5.4%)		166 (5.2%)		45 (5.5%)		60 (5.7%)	
Not Hispanic or Latino	4701 (93.1%)		2965 (93.2%)		759 (92.9%)		977 (93.2%)	
Unknown/patient declined	74 (1.5%)		50 (1.6%)		13 (1.6%)		11 (1.1%)	

### 2.2 Classifier Training

**Fig. 1:**
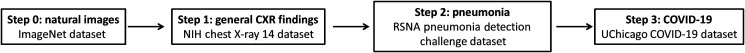
Flow chart of sequential transfer leaning diagram for ICU admission prediction of COVID-19 patients.

The DenseNet121 architecture was chosen for this study because of its success in the diagnosis of various diseases on CXR in previous publications.^40^^–^^42^ Instead of presenting the model with a random mixture of CXR examples to learn to detect COVID-19, a sequential transfer learning technique was employed to train the model on a sequence of gradually more specific and complex tasks to mimic the human learning process.^43^ First, a model pretrained on ImageNet^44^ with 1.2 million natural images was fine-tuned on the National Institutes of Health (NIH) ChestX-ray14 dataset to detect 14 common disease types.^44^^,^^45^ Then, the model was fine-tuned on the Radiological Society of North America Pneumonia Detection Challenge dataset, which has a high pneumonia prevalence, ∼24%, to detect evidence of pneumonia.^46^ The data for this pneumonia detection challenge can be accessed through the challenge website.^46^ The ground truth was provided by the radiologists at the Society for Thoracic Radiology by labeling pneumonia cases. Finally, the model was fine-tuned again on the training set of our COVID-19 dataset and then ultimately evaluated on the independent held-out test set in the task of intensive care prediction for COVID-19 patients, as conducted in our previous preliminary study.^47^ For the preprocessing, the images were down sampled to 256×256  pixels and gray-scale normalized. Images were randomly augmented by horizontal flipping, rotation of up to 8 deg and shifting by up to 10% of the image size. The model was trained with weighted cross-entropy loss function, Adam optimizer, and a batch size of 64 with an initial learning rate of 0.0001. Step decay on learning rate and early stopping were employed. The details regarding this cascade model training approach can be found elsewhere.^10^^,^^47^ The sequential transfer learning diagram for predicting ICU admission of COVID-19 patients is shown in Fig. 1. The dataset was randomly split at the patient level into 64% for training, 16% for validation, and 20% for testing using stratified sampling, holding the class prevalence for the least frequent outcome, i.e., intubation or ICU admission within 24 h, constant across all subsets. Dataset statistics and the prevalence of cases that required intensive care within 24, 48, 72, and 96 h after chest radiography exams are summarized in Table 2.

**Table 2:** Dataset statistics and the prevalence of cases that required intensive care within 24, 48, 72, and 96 h after chest radiography exams. The number of patients and images in each subset are listed.

Entire dataset		Overall	Training	Validation	Test
**Total**	Patient	5046	3181 (63.0%)	817 (16.2%)	1048 (20.8%)
	Image	8357	5347 (64.0%)	1338 (16.0%)	1672 (20.0%)
**ICU cases**		**Overall**	**Training**	**Validation**	**Test**
**24 h**	Patient	730 (14.5%)	468 (14.7%)	115 (14.1%)	147 (14.0%)
	Image	979 (11.7%)	626 (11.7%)	157 (11.7%)	196 (11.7%)
**48 h**	Patient	790 (15.7%)	505 (15.9%)	125 (15.3%)	160 (15.3%)
	Image	1104 (13.2%)	718 (13.4%)	172 (12.9%)	214 (12.8%)
**72 h**	Patient	801 (15.9%)	512 (16.1%)	126 (15.4%)	163 (15.6%)
	Image	1174 (14.0%)	772 (14.4%)	179 (13.4%)	223 (13.3%)
**96 h**	Patient	809 (16.0%)	519 (16.3%)	126 (15.4%)	164 (15.6%)
	Image	1222 (14.6%)	808 (15.1%)	185 (13.8%)	229 (13.7%)

### 2.3 Performance Evaluation

Performance was evaluated for the task of predicting the need for intensive care within 24, 48, 72, and 96 h after each CXR exam in the test set (1048 patients, 1672 CXR exams). Here, the classification performance for each label was evaluated using receiver operating characteristic (ROC) analysis with area under the proper binormal ROC curve (AUC) as the figure of merit.^48^^,^^49^ The 95% confidence intervals (CIs) of the AUC values were calculated by bootstrapping the posterior probabilities of the test set (5000 bootstrap samples).^50^ The statistical difference between the AUC values for different models was computed using ROCKIT software.^51^ Gradient-weighted class activation mapping (Grad-CAM) was generated to provide a visual explanation of the model’s classification.^52^ The second performance evaluation was performed by patient and involved the first CXR exam of each patient only (1048 patients, 1048 CXR exams). Here, time-to-event analysis^53^^,^^54^ was performed based on the AI/ML output for the task of predicting the need for intensive care within 96 h after the initial CXR exam. The median of the intensive care risk score (the AI/ML output) was used to divide the patient cohort into “high risk” and “low risk” subsets, and the corresponding hazard ratio was calculated. The third analysis involved post-hoc stepwise fitting of a linear regression model using the intensive care risk score, patient age, sex, race, ethnicity, and immunization status as initial variables to investigate whether variables other than the AI/ML output, i.e., the ICU/intubation risk score, were important for determining the patient prognosis within our test cohort. All reported performances pertain to the independent test set (1048 patients).

## 3 Results

**Fig. 2:**
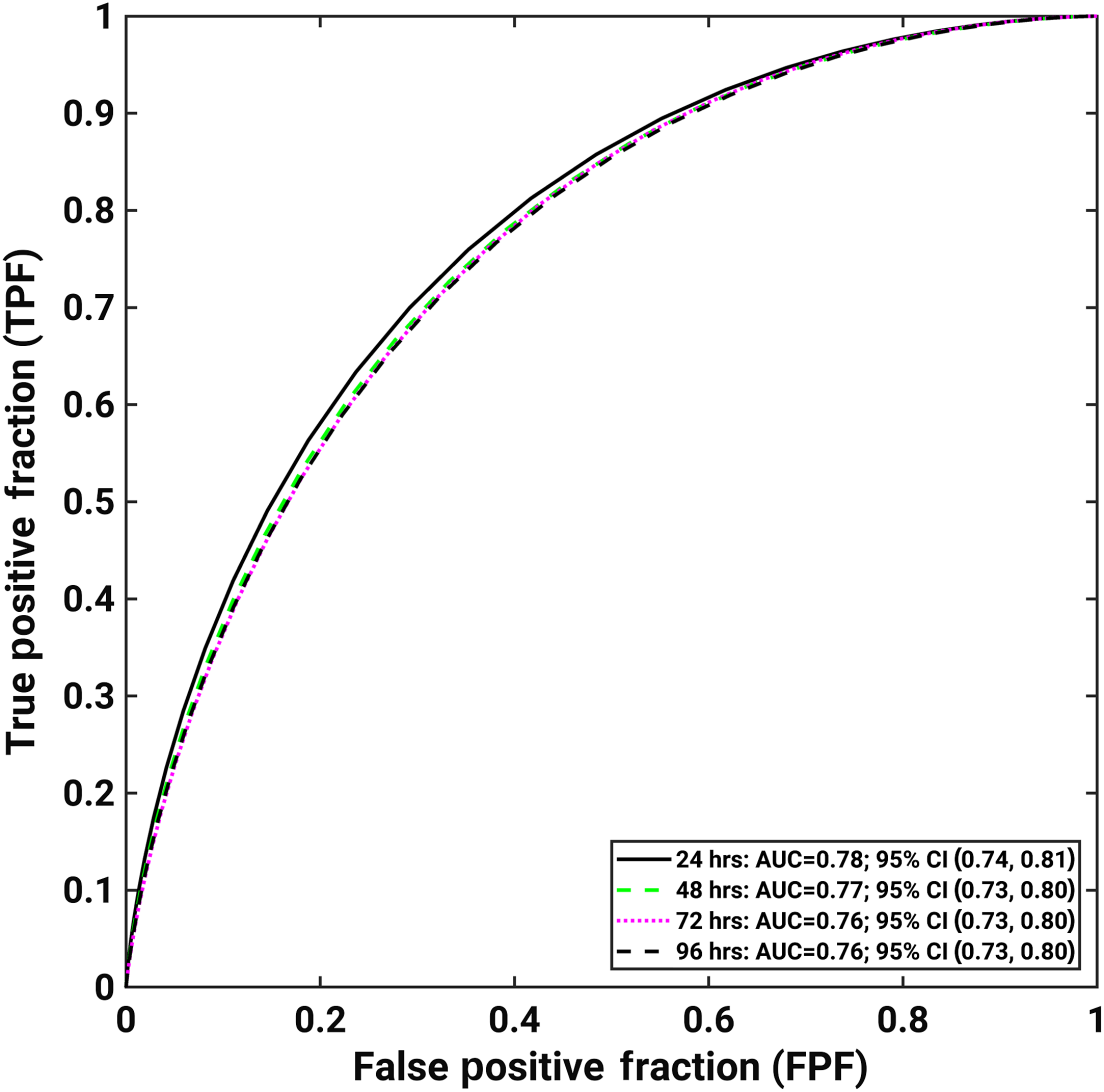
ROC curves for classification tasks requiring intensive care or not within 24, 48, 72, and 96 h from image acquisition. The legend gives the AUC with 95% CI for each task.

The ROC curves for predicting COVID-19 patients’ potential need for intensive care in 24, 48, 72, and 96 h in advance are shown in Fig. 2. We achieved an AUC (95% CI) of 0.78 (0.74, 0.81) when predicting ICU admission 24 h in advance, while also achieving promising performances in predictions made more in advance: 0.77 (0.73, 0.80), 0.76 (0.73, 0.80), and 0.76 (0.73, 0.80) when predicting ICU admission 48, 72, and 96 h in advance, respectively.

**Fig. 3:**
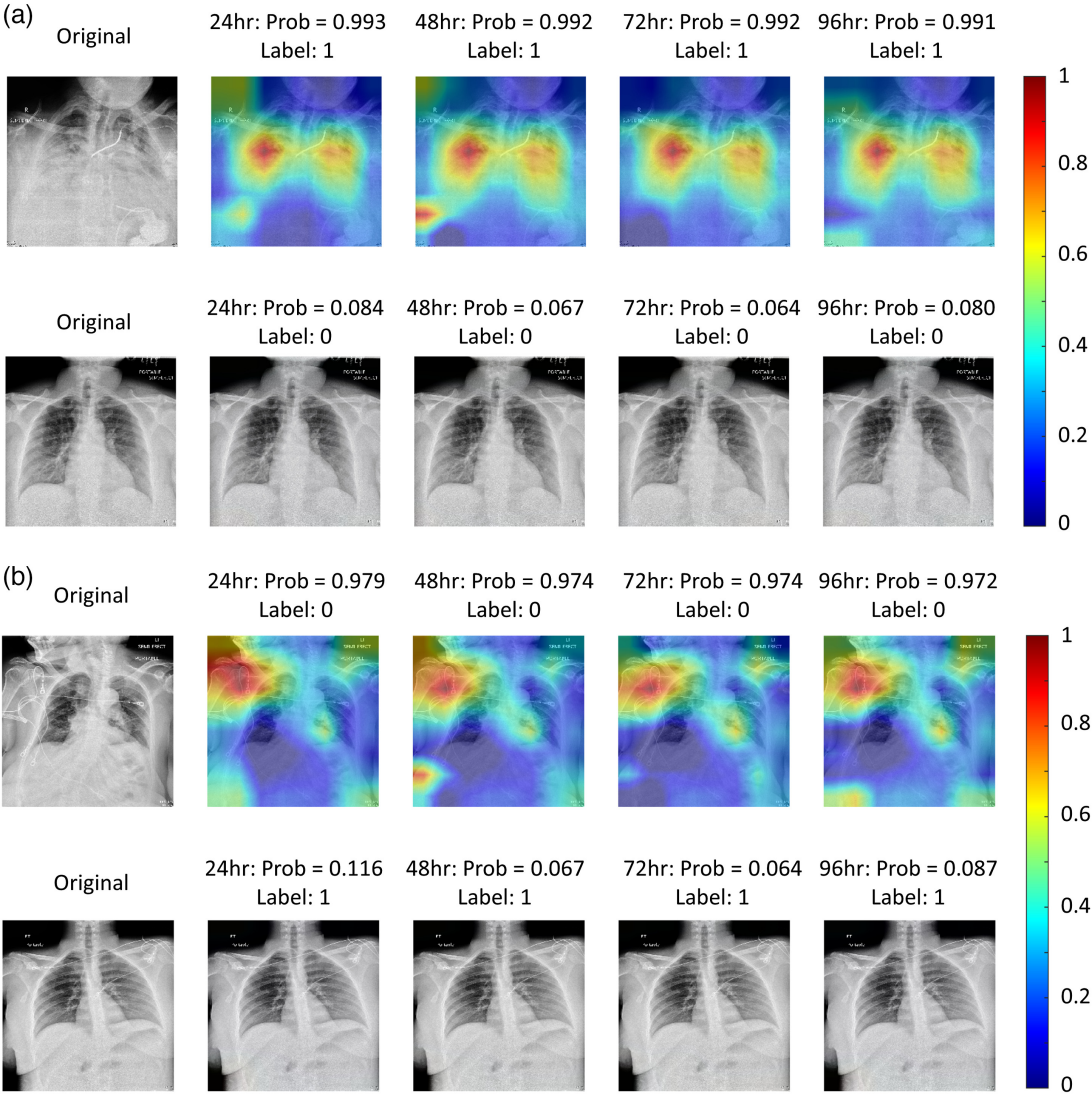
Example CXRs overlaid with their Grad-CAM heatmaps for prediction of the need for intensive care within 24, 48, 72, and 96 h, respectively, for instances (a) in which the AI/ML prediction was correct and (b) in which the output was incorrect. The probability is the model output for the likelihood of receiving intensive care scaled to 50% prevalence.^55^ The term “label” in the figure reflects the “ground truth” for the intensive care requirement: 1 for ICU admission/intubation and 0 for no ICU admission/intubation. (a) The patient in the top example was admitted into ICU within 4 h after image acquisition (true positive example). The patient in the bottom example did not require intensive care within 96 h after image acquisition (true-negative example). (b) The top is a false positive example and the bottom is a false negative example.

Figure 3(a) shows two examples, each with the original CXR image and the Grad-CAM heatmap from the last batch normalization layer of the model overlaid on the CXR image. The top row in Fig. 3(a) is from a COVID-19–positive patient who was admitted to ICU within 4 h following image acquisition. The bottom row in Fig. 3(a) is from a COVID-19–positive patient who did not receive intensive care within the 96 h after the CXR image was acquired, most likely due to a mild assessment of the likelihood of receiving intensive care. The predictions for intensive care within 24, 48, 72, and 96 h after CXR images agreed with the clinical assessment with both patients. The highlighted areas from the Grad-CAM heatmaps showed the abnormalities in the lungs indicating those areas of lung that had the most impact on the classification score, i.e., on the probability of COVID-19–positive patient to be admitted into ICU. This elevated Grad-CAM signal in the COVID-19 patient could be an indication of pneumonia and may be associated with the extent of ground glass/hazy opacities and consolidation of the lung area. Figure 3(b) shows two examples, the top row is a false positive example and the bottom one is a false negative example.

**Fig. 4:**
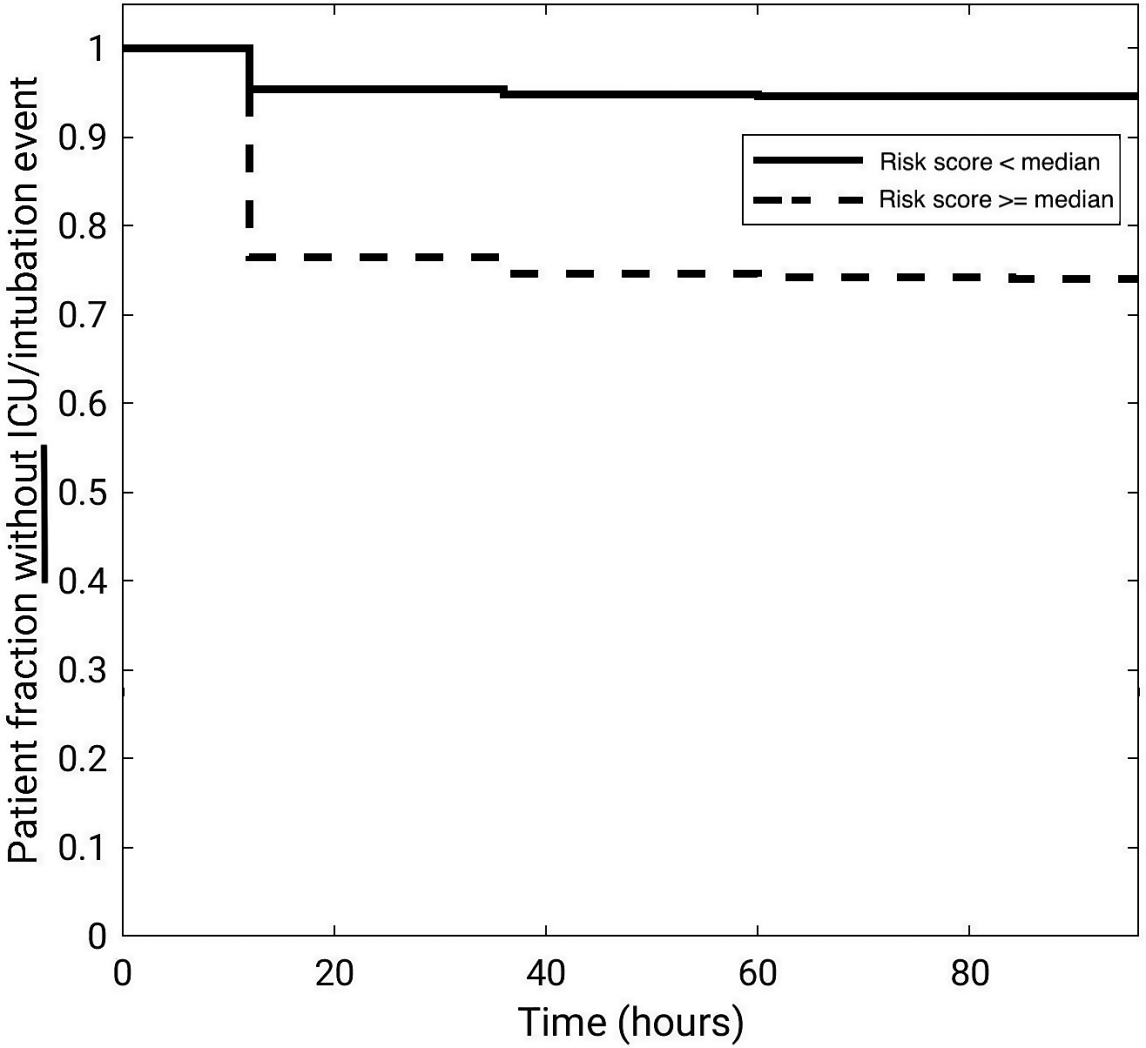
Time-to-event analysis for the need of intensive care within the 96-h time window after each patient’s first CXR exam. The time progression of the data is plotted at the midpoint of the time interval. For example, the patient fraction without ICU/intubation event at 24 h post-imaging is plotted at 12 h.

The time-to-event analysis demonstrated that the “high risk” subset of patients (the half of the cohort with a risk score larger than/equal to the median score) had a significantly increased risk of the need for intensive care than the “low risk” subset (the half of the cohort with a risk score lower than the median score, Table 3, Fig. 4). The hazard ratio was 0.22 [95% CI (0.16, 0.30); p-value<0.0001].

**Table 3:** The number of ICU admission/intubation events within the different time windows for the “high risk” and “low risk” patient subsets of the test set, i.e., for those patients receiving a risk score smaller than, or larger/equal to, the median score of the test cohort in its entirety.

ICU admission/intubation events			
Time window (h)	“High risk” cohort (N=524)	“Low risk” cohort (N=524)	Entire cohort (N=1048)
0 to 24	123 (23.5%)	24 (4.6%)	147 (14.0%)
24 to 48	10 (1.9%)	3 (0.6%)	13 (1.2%)
48 to 72	2 (0.4%)	1 (0.2%)	3 (0.3%)
72 to 96	1 (0.2%)	0 (0%)	1 (0.1%)
0 to 96	136 (26.0%)	28 (5.3%)	164 (15.6%)

In the stepwise fitting of a linear regression model using the intensive care risk score, patient age, sex, race, ethnicity, and immunization status as initial variables, the intensive risk score was selected first (p-value<0.0001), and patient sex was selected second (p-value=0.020), with the women being at a slightly lower risk of needing intensive care than the men in our cohort. All other variables failed to reach statistical significance and were not selected.

## 4 Discussion

In this work, we present a deep learning method that can predict the need for intensive care of COVID-19–positive patients using CXR images, where intensive care is defined as intubation and/or ICU care, i.e., a prognostic marker of COVID severity.

Note here, without fine-tuning, the AUCs of 0.72 (0.68, 0.76), 0.70 (0.67, 0.74), 0.70 (0.66, 0.73), and 0.70 (0.66, 0.73) were obtained when predicting ICU admission 24, 48, 72, and 96 h in advance, respectively. We observed statistically significant improved performance in predicting ICU admission between two schemes, with fine-tuning and without fine-tuning, 0.78 versus 0.72 [95% CI of ΔAUC (0.0200, 0.0941), p=0.0025]; 0.77 versus 0.70 [for 95% CI of ΔAUC (0.0305, 0.1038), p=0.0003]; 0.76 versus 0.70 [95% CI of ΔAUC (0.0305, 0.1011), p=0.0003]; and 0.76 versus 0.70 [95% CI of ΔAUC (0.0322, 0.1027), p=0.0002] for predicting ICU admission 24, 48, 72, and 96 h in advance, respectively. These results indicated that this sequential transfer learning strategy may be useful on improving the model performance.

A similar study by Shamout et al.^56^ predicted patient deterioration and achieved an AUC of 0.786 (0.745, 0.830) when using both clinical variables and imaging data and 0.738 (0.695, 0.785) when using CXR image data alone. Although a direct quantitative comparison with the existing approaches was not feasible due to the differences in the task definition and datasets, interestingly, our study, using imaging data alone, yielded a similar AUC to Shamout’s results incorporating both clinical and imaging data. Li et al.^16^ also reported a COVID-19 pulmonary disease severity model using CXR and achieved an AUC of 0.80 (95% CI 0.75 to 0.85) in prediction of subsequent incubation or death within 3 days of hospital admission. Others have investigated ICU admission prediction based on clinical characteristics alone. Zhao et al.^32^ built a prediction model for ICU admission based on clinical characteristics of COVID-19 patients. That risk score model yielded an AUC of 0.74 (0.63, 0.85) for predicting ICU admission. A similar study by Li et al.^30^ using only clinical variables achieved an AUC of 0.780 (0.760, 0.785) in ICU admission prediction with deep learning model, interestingly, our study achieved comparable performance using image data alone.

The potential clinical utility of our CXR imaging-based ICU admission/intubation risk score is further emphasized by both the presented time-to-event analysis and the fitted linear regression model. In the former, patients deemed to be “high risk” by our AI/ML model were almost five times as likely to require intensive care compared to those deemed “low risk.” In the latter, the linear regression model included only patient sex as contributing significantly to the prediction of the need for intensive care and coming second after the AI/ML predicted risk score. It should be noted; however, that for different patient cohorts, demographical characteristics may play a larger role since our institution serves a population with a demographic distribution that is different from those of the US census^57^ or CDC.^58^

The majority of previous publications using imaging data of COVID-19 patients focus on diagnosis rather than prognosis.^12^^,^^59^^–^^65^ While early and rapid diagnosis is crucial for highly infectious diseases, such as COVID-19, laboratory testing ability has largely advanced so that timely diagnosis by imaging is a lesser concern. Prognostic tasks are challenging but have substantial benefits including accurately triaging patients and forecasting demands on related hospitalization resources. An imaging-based model that can predict intensive care needs could potentially help to alleviate these challenges. We expect our CXR-based AI model could supplement prior AI studies, which only incorporated clinical variables, such as vital signs and laboratory tests or CT images,^56^^,^^66^^–^^68^ in the prognosis of COVID-19 patients.

Some cases were classified as false positive or false negative by the model and there are some factors that could have contributed to this. First, the influence from irrelevant regions on CXR images on the prediction of ICU admission status may contribute to the false positive cases. Incorporating lung region segmentation and cropping in the model could reduce false positive cases. Second, CXR images are a primary imaging modality for assessing the COVID-19 disease progression and pulmonary disease is the main complication associated with COVID-19 patients. However, some COVID-19 patients may have other non-pulmonary related comorbidities contributing to their deteriorating health and their ICU admission. These could cause a false negative prediction by the model. By incorporating both imaging and non-imaging data, including clinical variables and lab test results in the model could reduce the false negative and improve the model performance.

Our study has some limitations, which will be addressed in future work. First, we will expand the database to include more images as well as images from other institutions, so that we can assess the robustness of our approach. While we had access to patient demographics, clinical variables were not readily available. Thus, we will gather clinical variables as part of future investigations. AL/ML models combining imaging data with clinical variables to predicting ICU admission will be explored. We will also investigate the role of temporal analysis, taking advantage of previous and follow-up CXR exams of COVID-19 patients to evaluate disease progression. Finally, we did not compare the performance of our AI/ML model to clinician performance in predicting ICU admission from CXR. A reader study will be conducted to gather clinicians’ performance on this ICU prediction task and compare with the proposed model to access the potential clinical benefit of our model.

In summary, a deep learning CXR-based model was developed to predict patients’ risk of requiring intensive care for COVID-19 at 24, 48, 72, and 96 h post-imaging. Overall, our findings show the promise of AI-assisted medical image analysis in COVID-19 prognostic task, which bear the potential to play an important role in supporting clinical decision-making especially in situations of limited resources. Our proposed model may be potentially useful for efficient patient triage and for low resourced regions that need to prioritize care, knowing who to treat immediately during a pandemic. This work has the potential to support both clinical decision-making and resource management.

## Data and Code Availability Statement

The data used for this manuscript, including CXR are not publicly available due to patient privacy and data sharing agreements.
